# Achievement of Room Temperature Superelasticity in Ti-Mo-Al Alloy System via Manipulation of ω Phase Stability

**DOI:** 10.3390/ma15030861

**Published:** 2022-01-23

**Authors:** Naoki Nohira, Wan-Ting Chiu, Akira Umise, Masaki Tahara, Hideki Hosoda

**Affiliations:** Institute of Innovative Research (IIR), Tokyo Institute of Technology, 4259 Nagatsuta-cho, Midori-ku, Yokohama 226-8503, Japan; chiu.w.aa@m.titech.ac.jp (W.-T.C.); umise.a.aa@m.titech.ac.jp (A.U.); tahara.m.aa@m.titech.ac.jp (M.T.)

**Keywords:** Al addition, ω phase, superelasticity, room-temperature superelasticity, shape memory effect, Ti-Mo-Al

## Abstract

The achievement of room-temperature (RT) superelasticity in a Ti-Mo-Al ternary alloy system through the addition of a relatively high concentration of Al to manipulate the phase stability of the ω phase is realized in this study. The composition of the Ti-6 mol% Mo (Ti-11.34 mass% Mo) alloy was designated as the starting alloy, while 5 mol% Al (=2.71 mass% Al) and 10 mol% Al (=5.54 mass% Al) were introduced to promote their superelastic behavior. Among the alloys, Ti-6 mol% Mo-10 mol% Al alloy, which was investigated for the very first time in this work, performed the best in terms of superelasticity. On the other hand, Ti-6 mol% Mo and Ti-6 mol% Mo-5 mol% Al alloys exhibited a shape memory effect upon heating. It is worth mentioning that in the transmission electron microscopy observation, ω phase, which appeared along with β-parent phase, was significantly suppressed as Al concentration was elevated up to 10 mol%. Therefore, the conventional difficulties of the inhibited RT superelasticity were successfully revealed by regulating the number density of the ω phase below a threshold.

## 1. Introduction

Shape memory alloys (SMAs) have been extensively investigated owing to their functional properties, such as shape memory effect (SME) and superelasticity. In particular, the β-Ti-based SMAs are considered as promising candidates for the biomedical and biomaterial communities due to the aforementioned functionalities (i.e., SME and superelasticity) and good biocompatibility [1,2].

In Ti alloys, additives are classified into α (hcp)-stabilizing and β (bcc)-stabilizing elements. Al, known as a typical one, is a standard element for the calculation of Al-equivalent as other α-stabilizing elements are used, while Mo is also used for the calculation of Mo-equivalent [3]. Therefore, Ti-Mo-Al alloy, which contains Al, a typical α-stabilizing element and Mo, a typical β-stabilizing element, is a basic and standard model for Ti-alloys, and is a crucial system for fundamental research of Ti-alloys. The achievement of superelasticity in this Ti-Mo-Al basic model, which is considered as a prototype of SMA, could be a guideline for the development of various types of other β-Ti-based SMAs via the equivalent calculations. The β-type Ti-Mo-Al system was thus prepared in this study for studying its superelasticity at room temperature (RT).

The SME of β-Ti-based alloys originates from phase transformation between β (bcc) parent and α″ (orthorhombic) martensite phases. The Lattice deformation strain of Ti-Mo alloys between β and α″ phases of about 9.5% is comparable to well-known Ti-Ni alloys even when Mo was introduced to lower martensitic transformation (MT) temperature to RT [4]. Therefore, SME of Ti-Mo-based alloys has been widely investigated [5,6,7,8]. Our group also succeeded in developing RT superelasticity via MT temperature regulation by alloying Zr and Sn to Ti-3 mol% Mo specimens [9]. Besides Ti-Mo alloys, the SME of Ti-Cr-Al [10] and Ti-Ta-Al [11] as well as superelasticity of Ti-Nb-Al [12,13,14] containing Al, have been explored.

Concerning Ti-Mo-Al systems, Sasano et al. studied SME of Ti-12Mo-3Al (mass%) alloy [15], followed by systematic studies with various Mo and Al concentrations. It was claimed that SME was obtained in some Ti-5Mo-(6–7)Al (mol%) and Ti-6Mo-(3–8)Al (mol%) alloys [16]. Nevertheless, the Al amount for investigations of SME and MT in these studies was limited to 3–8 mol% (1.5–4.5 mass%).

Ijaz et al. discovered that stress hysteresis (Δ*σ*) of superelastic Ti-Nb-Mo alloys was alleviated via Sn addition, which suppresses formations of the ω phase [17]. Here, Δ*σ* is the difference between the stress for inducing MT of β phase (*σ*_β-α″_) during loading and the stress for finishing of reverse MT (*σ*_α″-β_) during unloading. In certain cases, reverse MT is not triggered even when applied stress is fully released in reverse MT due to a large Δ*σ*, whereby superelasticity deteriorates or vanishes. To achieve superelasticity, Δ*σ* must be reduced sufficiently for the commencement of reverse MT. It is also known that Al also mitigates formations of ω phases in a similar manner as Sn [10,18,19,20]. Williams et al. studied the effects of Al on formations of the ω phase in Ti-6 mol% Mo alloys and stated that volume fraction (VF) of the ω phase was reduced with Al [21].

Some studies revealed the SME of the Ti-Mo-Al alloys [15,16]; however, there is still a lack of research regarding superelasticity of this system at RT. For various applications, it is critical to put both SME and superelasticity into practice in the most fundamental β-Ti-based SMAs, such as the Ti-Mo-Al in this study, since it can be a potential tool for the preparation of various types of the β-Ti-based SMAs via the Mo- and Al-equivalent calculations, as mentioned. Additionally, judging from the literature, it was concluded that by means of the already-studied Al composition range (i.e., less than 8 mol%) in Ti-Mo-Al alloys, it is insufficient to inhibit the formation of ω phase; consequently, superelasticity was not unveiled due to a large Δ*σ*. Therefore, it is assumed that relatively high Al addition is necessary for the development of RT superelasticity in the Ti-Mo-Al system. Based on this assumption, RT superelasticity is expected to be imposed in Ti-Mo-Al alloys as Al concentration is beyond 8 mol%, which is higher than those in the literature.

In this work, to verify this hypothesis about the aforementioned Al addition concentration, the Ti-6 mol% Mo alloy, which allows the formation of the β phase at RT through quenching followed by solution-treatment, was chosen as the prototype specimen. Furthermore, specific Al concentrations of 5 mol% (i.e., within the range studied by literature) and 10 mol% (i.e., out of the literature range) were designed, respectively, to certify the speculation. The correlation between deformation behaviors and formations of ω phase in Ti-Mo-Al alloys was further investigated.

## 2. Materials and Methods

High purity Ti (99.99%), Mo (99.9%), and Al (99.99%) were used for the preparation of Ti-6Mo-(0, 5, and 10) Al (mol%) alloys. Ingots of 8 g were fabricated by arc-melting in an Ar-1 mol% H_2_ atmosphere. Specimens are abbreviated as 0 Al, 5 Al, and 10 Al, unless otherwise stated. Arc-melted ingots were sealed in quartz tubes under an Ar atmosphere, homogenized at 1273 K for 7.2 ks, quenched in iced-water, and cold-rolled into sheets. Specimens used for measurements were cut by electro-discharge machining, solution-treated at 1273 K for 1.8 ks in Ar atmosphere, followed by iced-water quenching.

For phase identification, X-ray diffraction (XRD; X′Pert-PRO-MPD, Malvern PANalytical, Malvern, UK) measurements were conducted by CuKα radiation within 2*θ* = 20–120° at RT (295 ± 2 K). Scanning electron microscopy (SEM; SU5000, Hitachi High-Tech Corporation, Tokyo, Japan) and transmission electron microscopy (TEM; JEM-2100, JEOL Ltd., Tokyo, Japan) were undertaken with acceleration voltages of 15 kV and 200 kV, respectively. Prior to TEM observations, thin foil specimens were processed by twin-jet polishing. Tensile tests were performed by an Instron universal testing machine (Autograph AG-Xplus 5 kN, Shimadzu Corporation, Kyoto, Japan) on 20 mm (length) × 2 mm (width) × 0.2 mm (thickness) specimens at RT. The tensile tests were conducted along the rolling direction of specimens at a strain rate of 8.3 × 10^−4^ s^−1^ and specimens were unloaded after approximately 4% strain deformation. Those specimens did not perform superelastic recovery, were subjected to heat-treatments up to 500 K ± 20 K after unloading, and shape recoveries were recorded to investigate SME.

## 3. Results and Discussion

**Figure 1:**
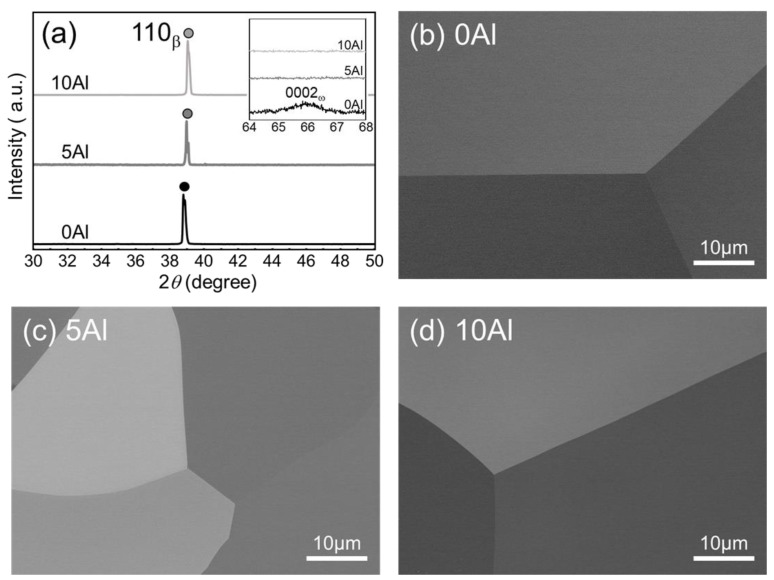
(**a**) XRD profiles of 0Al, 5Al, and 10Al solution-treated alloys (magnified X-ray diffraction patterns for three specimens from 2*θ* = 64° to 68° are inserted in the top-right corner of (**a**) to reveal the diffraction peaks of ω phase). SEM images of (**b**) 0Al, (**c**) 5Al, and (**d**) 10Al.

All alloys possessed the β phase in XRD profiles (Figure 1a), and as shown in the inset, a small peak was observed in 0Al, which could be indexed as ω phase. Equiaxed grains of about 200 μm were observed in OM images (Figure S1 in the Supplementary Materials) and no secondary phase was discerned in SEM images (Figure 1b–d).

**Figure 2:**
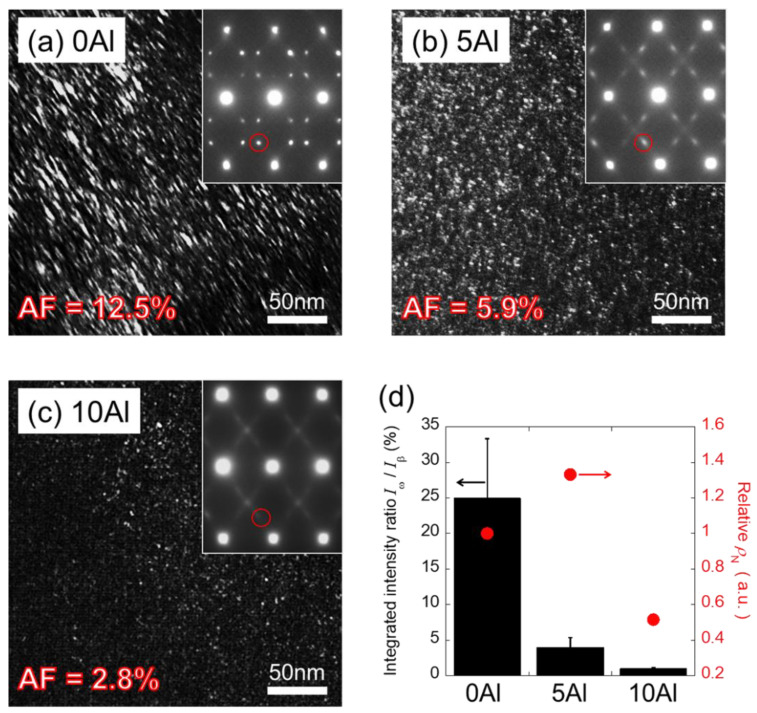
TEM DF images and corresponding SADPs (inserted to upper-right corner) with a [110]_β_ zone axis of (**a**) 0Al, (**b**) 5Al, and (**c**) 10Al. Red circles in the insets of (**a**–**c**) indicate ω_ath_ diffractions. (**d**) The ratio of *I*_ω_/*I*_β_ of ω_ath_ spot intensity to β spot intensity (black bars with error bars) and relative number density (*ρ*_N_) of ω_ath_-particles (red solid circles) for each alloy. Note that the relative *ρ*_N_ for each alloy is normalized by taking 0Al as a standard.

Dark-field (DF) images along with corresponding selected area diffraction patterns (SADPs) from the [110]_β_ direction are shown in Figure 2 and a fine secondary phase was identified. Besides diffraction spots of the β phase, notwithstanding the Al amount, spots of the ω phase were further observed at 1/3 and 2/3 positions along the <112>*_β_ direction in all SADPs. This ω phase was recognized as athermal ω (ω_ath_), whose area fraction (AF) and size were evaluated by ImageJ 1.51k. Not surprisingly, the highest AF of 12.5% possessing a major axis of approximately 6.9 nm and a minor axis of approximately 2.3 nm of ω_ath_ phase, was found in 0Al (Figure 2a). AF of 5.9% with an average size of 3.3 nm and AF of 2.8% with an average size of 3.1 nm of the ω_ath_ phase were found in 5Al (Figure 2b) and 10Al (Figure 2c), respectively. It was thus concluded that the size and AF of the ω_ath_ phase could be diminished with Al introduction. Moreover, spots of the ω_ath_ phase in 0Al was observed distinctly and sharply; conversely, spots turned diffuse and faint with the Al introduction (i.e., 5Al and 10Al), indicating that SADPs results agreed well with DF images.

Since it is crucial to take specimen thickness into account, the total amount of ω_ath_ phase was further estimated by the *I*_ω_/*I*_β_ measure of the spot intensity ratio of the ω_ath_ phase (*I*_ω_) to β phase (*I*_β_) (Figure 2d), which was calculated from the integrated intensity profiles of SADPs along <112>*_β_ [22]. *I*_ω_/*I*_β_, which is equivalent to VF, are approximately at 25%, 4%, and 1% for 0Al, 5Al, and 10Al, respectively. The subsided spot intensity of ω_ath_ phase, suggesting the diminished VF of the ω_ath_ phase originated from elevated Al. A remarkable suppression of the ω_ath_ phase was especially found in 10Al (i.e., the highest Al) and the results are consistent with literature [20,21]. The quantification of the SADPs can be difficult; nevertheless, it is apparent that the diffraction spots of the ω_ath_ phase gradually turned weak and diffuse from 0Al to 10Al.

Precipitates of the ω_ath_ phase inhibiting stress-induced martensitic transformation (SIMT), increased the stress for β/α″ interface migration in a solid-solution strengthening-like manner. Accordingly, intensified stress is reasonably speculated to correlate positively to number of pinning at the interface. First, it is assumed that ω_ath_-particles are homogeneous in size and low-composition dependent in shape. Second, *I*_ω_/*I*_β_ divided by the volume of one ω_ath_-particle is proportional to the number density (*ρ*_N_). A relative *ρ*_N_, which is normalized by serving 0Al as the standard, is used and shown in Figure 2d. The highest and lowest relative *ρ*_N_ were found of 1.3 and 0.5 for 5Al and 10Al, respectively. It is thus concluded that size and *I*_ω_/*I*_β_ (i.e., VF) were reduced with Al; by contrast, relative *ρ*_N_ does not show specific dependency.

**Figure 3:**
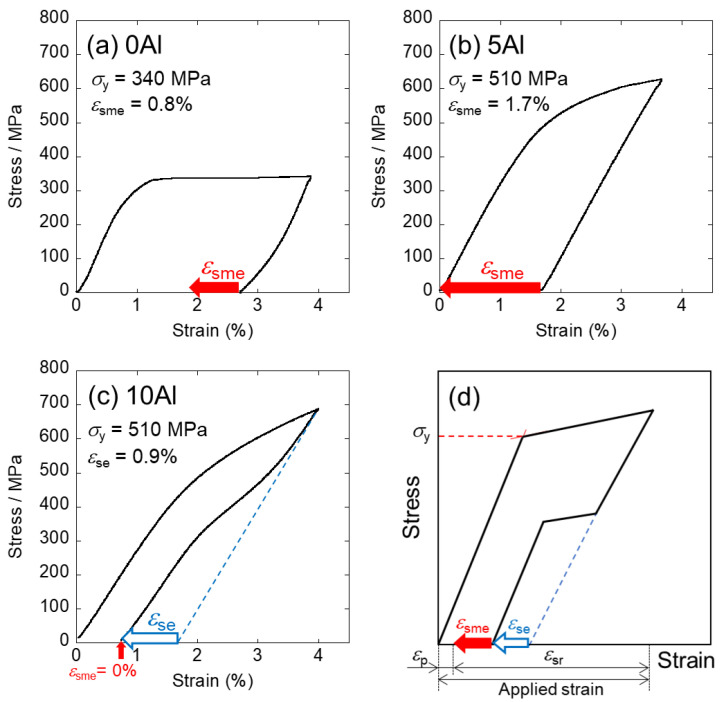
Stress-strain curves of (**a**) 0Al, (**b**) 5Al, and (**c**) 10Al. (**d**) Definition of each term. (Open blue arrow symbols: strain originates from superelasticity during unloading (*ε*_se_); solid red arrow symbols: strain brought by SME upon heating (*ε*_sme_); *ε*_sr_: strain of overall shape recovery; *ε*_p_: strain by plastic deformation; *σ*_y_: yielding stress; applied strain = *ε*_sr_ + *ε*_p_).

Stress-strain curves and definitions are shown in Figure 3. First, yielding stress (*σ*_y_) of 0Al was found at 340 MPa and the applied strain almost remained after unloading (Figure 3a). About 1/3 residual strain (i.e., 0.8%) of shape recovery took place upon heating due to SME (*ε*_sme_). These results agreed well with Ti-6 mol% Mo and Ti-11 mass% Mo (= Ti-5.81 mol% Mo) alloys [23,24]. Additionally, Ti-11 mass% Mo alloy possessing proximate composition to 0Al was reported to undergo plastic deformation mainly by twinning deformation of the β phase [25,26] and was accompanied by SIMT simultaneously in the early stage [27,28,29,30]. Moreover, Oka et al. also reported that a slight SIMT occurred in 10% cold-rolled Ti-11 mass% Mo alloy [31]. It is thus concluded that twinning of the β phase is the major mechanism during deformation, while a minor amount of SIMT to the α″ phase, which went along with it, led to shape recovery upon heating. Furthermore, since merely slight work-hardening was found in 0Al, *σ*_y_ is considered to be equivalent to the stress for SIMT (*σ*_SIMT_). Note that slight pseudoelastic recovery during unloading was attributed to twinning pseudoelasticity caused by stress-induced martensitic variants, which is often seen in Ti-Mo-based SMAs and others.

Second, in 5Al (Figure 3b), *σ*_y_ was identified at approximately 510 MPa, which is higher than 0Al. By contrast with 0Al, 5Al performed neither pseudoelastic nor superelastic recovery; instead, the elastic recovery was merely found during unloading. It is worth mentioning that followed by unloading, 100% shape recovery was realized upon heating performing a perfect SME. Accordingly, it is rational to infer that *σ*_y_ corresponds to *σ*_SIMT_ and these results are supported by literature [15].

Lastly, unexplored 10Al (Figure 3c), whose *σ*_y_ was at approximately 510 MPa, is similar to 5Al; nevertheless, a larger shape recovery than the amount brought by merely elastic recovery was recognized during unloading. This indicated that superelasticity was successfully revealed in 10Al showing a superelastic recovery strain *ε*_se_ of 0.9% while no SME was found in 10Al upon heating and the residual strain remained. It is considered that the residual strain could be ascribed to slip deformation, which originates from dislocation motions, and arises along with SIMT during loading. The RT superelasticity of 10Al was practiced for the very first time in this study. Judging from the results, *σ*_y_ of 10Al is surmised to be both a critical stress for slip deformation and *σ*_SIMT_. In short, *σ*_y_ of all specimens was accompanied with SIMT during loading in this study.

**Figure 4:**
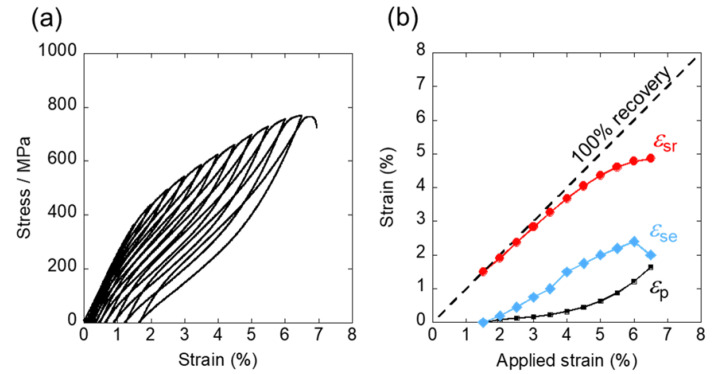
(**a**) Stress-strain curves of 10Al obtained from the cyclic loading-unloading tensile test. (**b**) Plot of various strains (overall shape recovery strain (*ε*_sr_), transformation strain of superelasticity (*ε*_se_), and residual plastic strain (*ε*_p_)) as a function of applied strain.

For further analysis of superelasticity of the 10Al, the cyclic loading-unloading tensile test was performed. Specimens were subjected to 1.5% stain in the first cycle followed by repeated 0.5% strain per cycle until they fractured. The stress-strain curves via the cyclic loading-unloading tensile test are shown in Figure 4a. The overall shape recovery strain (*ε*_sr_), transformation strain of superelasticity (*ε*_se_), and residual plastic strain (*ε*_p_) of each cycle, which were evaluated from Figure 4a, are plotted as a function of applied strain in Figure 4b. It was found that *ε*_sr_ and *ε*_se_ increased linearly up to an applied strain of 6% before decreasing. The maximum *ε*_se_ was 2.4% at an applied strain of 6%, and the maximum *ε*_sr_ reached 4.8%. The obtained *ε*_se_ in this 10Al surpassed that of 2.3% *ε*_se_ of the greatly investigated Ti-26Nb (mol%) alloy [32]. It is expected that further manipulation of the alloy composition and/or heat-treatment could develop highly practical SMAs with promoted superelastic strain. Our research group is still working on the development of other related alloys, and improvements of the β-Ti-based SMAs will be published in future.

Given that 0Al and 5Al performed SME while 10Al exhibited superelasticity, reverse MT start temperatures (*A*_s_) are deduced to be lower than the heating temperature of 500 K and RT of around 300 K, respectively. This suggests that *A*_s_ was reduced by more than 200 K via 10% Al addition. The reduced amount in this study agreed well with that 25–40 K/mol% Al claimed in literature [12,33,34].

Firstly, from transformation stress and temperature points of view, generally, the *σ*_β-α″_ of SMAs follows Clausius-Clapeyron equation and increases linearly with temperature difference between MT and operation temperatures. Assuming that forward MT temperature also decreases with Al in the similar manner of reverse MT temperature, the lowest *σ*_β-α″_ would appear in 0Al and increase with Al. However, composition dependence of *σ*_β-α″_ in this study did not show such a trend. Secondly, from a phase equilibrium point of view, while taking *A*_s_ as phase equilibrium temperature of β phase and α″ phase, stable phase at RT for 0Al and 5Al should be the α″ phase. Despite the speculation, apparent phases were β + ω_ath_. Therefore, finally, it is rational to consider that β phase was “quenched-in” by formations of the ω_ath_ phase. According to the inferences, it is necessary to take the suppression of SIMT by the ω_ath_ phase into account. It is expected that suppression of phase transformation increases with the ω_ath_ phase amount resulting in high *σ*_SIMT_.

A small amount of the ω_ath_ phase (i.e., small *I*_ω_/*I*_β_) but high *ρ*_N_ was found in 5Al, whose *σ*_β-α″_ at 510 MPa was 170 MPa higher than 340 MPa of 0Al, indicating that suppression of SIMT greatly depends on *ρ*_N_. Not surprisingly, 10Al possessing both a small amount and low *ρ*_N_ of the ω_ath_ phase performed superelasticity. This could be attributed to the least suppressed SIMT leading to a limited increment of *σ*_β-α″_. It was concluded that to achieve RT superelasticity, it is crucial to suppress both the amount and *ρ*_N_ of the ω_ath_ phase. Based on our results, keeping *ρ*_N_ below a threshold is especially an effective strategy. Therefore, introducing additive elements which sufficiently suppress the ω_ath_ phase could be practicable; moreover, as mentioned previously, *A*_s_, which is less affected by ω_ath_ phase than *M*_s_, is also a critical factor.

**Figure 5:**
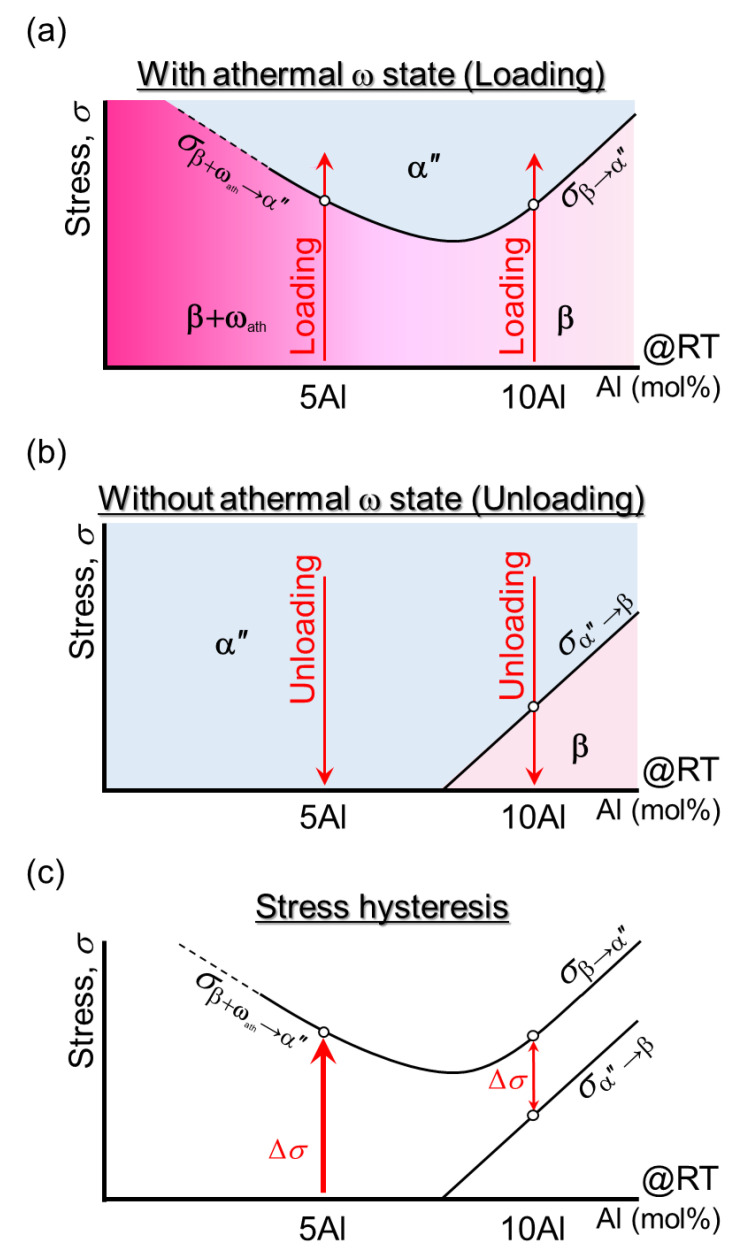
Illustrations of the effect of Al concentration on (**a**) phase stability during loading w/ω_ath_ phase, (**b**) phase stability during unloading w/o ω_ath_ phase, and (**c**) stress hysteresis of Ti-6Mo-Al alloys.

Based on the results, the effect of Al on *σ*_β-α″_ and *σ*_α″-β_ are summarized and illustrated in Figure 5 and Table S1. In Figure 5a, *σ*_β-α″_ deviates from the linear relationship showing an inflection point as the composition goes from high Al (i.e., 10Al) to low Al (i.e., 5Al), which contains high *ρ*_N_ and an increment of *σ*_β-α″_ was thus imposed. On the other hand, during unloading, the effect from the ω_ath_ phase, which suppressed reverse SIMT, is limited; therefore, a linear relationship was constructed (Figure 5b). A summary of stress hysteresis (Δ*σ*) based on the superimposing of loading (Figure 5a) and unloading (Figure 5b) results is illustrated in Figure 5c. Obviously, significant reduction of Δ*σ* achieved by 10Al, was found at approximately 180 MPa (Figure 3c).

The phase constituents and phase transformations are summarized in Table 1. There are two possible reasons for achieving RT superelasticity via 10 mol% Al addition. First, the transformation temperature of 10Al was lower than 5Al and the stable phase at RT thus turned into β phase. Second, both the amount and *ρ*_N_ of the ω_ath_ phase were greatly suppressed; accordingly, Δ*σ*, which was mitigated to about 180 MPa, enabled reverse SIMT at RT. In brief, for obtaining RT superelasticity, an Al amount up to 10 mol% is a crucial prerequisite.

In 5Al, by contrast, despite a small amount of the ω_ath_ phase, Δ*σ* was deteriorated by high *ρ*_N_. Whether superelasticity could be imposed by simply introducing Mo for reducing *A*_s_ below RT at 5 mol% Al or not, is considered by making the following presumptions. According to the results, it is known that *σ*_β-α″_ of 5Al is 510 MPa and Δ*σ* at around 700 MPa is also estimated (Figure 3b). It could also be inferred that, like 10Al, β phase would be stable at RT as *A*_s_ is 100 K lower than current *A*_s_. According to literature concerning metastable β-Ti alloys, the slope of the Clausius-Clapeyron relation (*dσ*_β-α″_/*dT*) is 3–5 MPa/K [35,36,37]. By considering the minimum of 3 MPa/K, it is deduced that transformation temperature of 100 K is reduced while *σ*_β-α″_ of 300 MPa is increased. Consequently, *σ*_β-α″_ would be at approximately 810 MPa via Mo introduction.

Generally, the *σ*_y_ of solution-treated Ti-alloys is less than 600 MPa, while the average critical stress for slip (*σ*_CSS_) of Ti-Mo-Sn-Zr alloys exhibiting RT superelasticity is 634 MPa [9], and Ti-20Zr-12Nb-2Sn alloy was found in a relatively high *σ*_CSS_ of approximately 800 MPa until very recently [38]. Therefore, MT is not induced in alloys possessing such a high *σ*_β-α″_; instead, only plastic deformation takes place. Specifically, both SME and superelasticity disappear. This thus explains well the vanishing superelasticity by merely regulating MT temperature via Mo addition without taking the ω_ath_ phase into consideration. Additionally, since many Ti-based superelastic alloys performed Δ*σ* less than 200 MPa [9,17], suppressing Δ*σ* to less than 200 MPa for revealing superelasticity is required; hence, Al addition up to 10 mol%, which possessed Δ*σ* of 180 MPa, is a crucial prerequisite.

## 4. Conclusions

In summary, size, amount, and *ρ*_N_ of the ω_ath_ phase were reduced to such an extent that the Δ*σ* in Ti-Mo-Al alloys was as low as 180 MPa by increasing Al to 10 mol%, which was beyond already studied samples and has not been explored. Meanwhile, RT superelasticity could be realized by lowering its *A*_s_ below RT. Based on equivalent calculations, additions greater than 6 mol% Mo-equivalent and 10 mol% Al-equivalent would perform RT superelasticity. The Mo-equivalent corresponds to 8.4 mol% Cr, 5.5 mol% Co, and 3.9 mol% Fe of typical β-stabilizing elements. Via precise composition manipulation, advancing unexplored RT superelastic alloys is foreseen.

## Figures and Tables

**Table 1:** The phase constituents and phase transformations at (a) quenched-in, (b) intrinsically stable (w/o ω_ath_ phase), (c) loading w/ω_ath_ phase, and (d) unloading w/o ω_ath_ phase states.

Alloy	(a) Quenched-In State	(b) Phase Stability	(c) Loading	(d) After Unloading
0Al	β + ω_ath_	α″	β + ω_ath_ → β_twin_ (+ α″)	β_twin_ (+ α″)
5Al	β + ω_ath_	α″	β + ω_ath_ → α″	α″
10Al	β (+ ω_ath_)	β	β → α″	β

## Data Availability

All data contained within the article.

## Supplementary Materials

The following supporting information can be downloaded at: https://www.mdpi.com/article/10.3390/ma15030861/s1, Figure S1: OM images of (a) 0Al, (b) 5Al, and (c) 10Al. Table S1: Summary of the (a) area fraction (AF) of ω_ath_ phase, (b) diameter of ω_ath_ phase, (c) intensity ratio of ω_ath_ phase to β phase *I*_ω_/*I*_β_, and (d) relative number density (*ρ*_N_).
