# Supplementary material for: Achievement of Room Temperature Superelasticity in Ti-Mo-Al Alloy System via Manipulation of ω Phase Stability
Source: Materials (Basel). 2022 Jan 23;15(3):861. doi: 10.3390/ma15030861 (PMC8836841; doi:10.3390/ma15030861)
Supplement: Supplementary file 1 [file materials-15-00861-s001.zip › materials-1563857-supplementary.pdf]

materials

Supplemental Materials

Achievement of Room Temperature Superelasticity in Ti–Mo–Al Alloy System via Manipulation of ω Phase Stability

Naoki Nohira *, Wan–Ting Chiu, Akira Umise, Masaki Tahara and Hideki Hosoda *

Institute of Innovative Research (IIR), Tokyo Institute of Technology, 4259 Nagatsuta–cho, Midori–ku, Yokohama 226–8503, Japan; chiu.w.aa@m.titech.ac.jp (W.-T.C.); umise.a.aa@m.titech.ac.jp (A.U.); tahara.m.aa@m.titech.ac.jp (M.T.)
* Correspondence: nohira.n.aa@m.titech.ac.jp (N.N.); hosoda.h.aa@m.titech.ac.jp (H.H.)

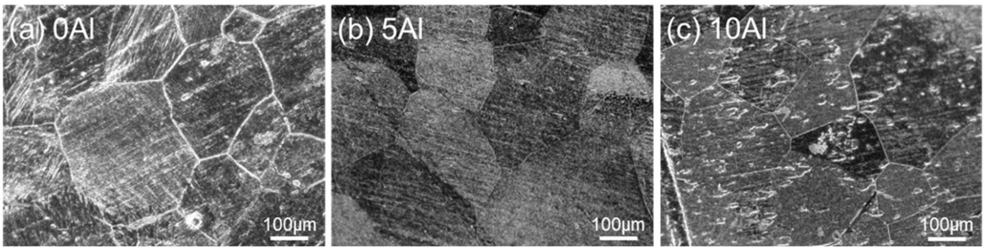

Figure S1. OM images of (**a**) 0Al, (**b**) 5Al, and (**c**) 10Al .

Table S1. Summary of the (a) area fraction (AF) of ω_{ath}-phase, (b) diameter of ω_{ath}-phase, (c) intensity ratio of ω_{ath}-phase to β-phase I_ω/I_β, and (d) relative number density (ρ_N).

	**(a) Area fraction (%) [AF]**	**(b) Diameter (nm) [D]**	**(c) Intensity ratio [I_ω/I_β]***	**(d) Relative number density [ρ_N]**
**0Al**	12.5	Major-axis: 6.9<br>Minor-axis: 2.3	25	1.0 (Standard)
**5Al**	5.9	3.3	4	1.3 (Normalized)
**10Al**	2.8	3.1	1	0.5 (Normalized)
**Criterion**	DF image	DF image	SADP**	$\frac{(I_\omega/I_\beta)}{Volume\ of\ 1\ \omega_{ath}\ particle}$

* Volume is considered via the integrated intensity profiles of SADPs; hence, the amount of intensity ratio is also known as “Volume Fraction (VF)”.

** Integrated intensity profiles of SADPs.

-Note that ( ) represents units and [ ] indicates symbols.

Citation: Nohira, N.; Chiu, W.-T.; Umise, A.; Tahara, M.; Hosoda, H. Achievement of Room Temperature Superelasticity in Ti-Mo-Al Alloy System via Manipulation of ω Phase Stability. *Materials* **2022**, *15*, 861. https://doi.org/10.3390/ma15030861

Academic Editor: Andrey Belyakov

Received: 4 January 2022
Accepted: 17 January 2022
Published: 23 January 2022

Publisher's Note: MDPI stays neutral with regard to jurisdictional claims in published maps and institutional affiliations.

Copyright: © 2022 by the authors. Licensee MDPI, Basel, Switzerland. This article is an open access article distributed under the terms and conditions of the Creative Commons Attribution (CC BY) license (https://creativecommons.org/licenses/by/4.0/).
